# Initiative on Rare and Undiagnosed Disease in Japan

**DOI:** 10.31662/jmaj.2021-0003

**Published:** 2021-04-08

**Authors:** Yuji Takahashi, Hidehiro Mizusawa

**Affiliations:** 1 Department of Neurology, National Center Hospital, National Center of Neurology and Psychiatry, Tokyo, Japan; 2 National Center of Neurology and Psychiatry, Tokyo, Japan

**Keywords:** IRUD, diagnostic and research scheme, whole exome sequencing, novel causative genes/disease entities, data sharing, international collaboration

## Abstract

The Initiative on Rare and Undiagnosed Diseases (IRUD) has established a unified all-Japan diagnostic and research scheme for rare and undiagnosed diseases covering the entire geographic areas and specialty/subspecialty fields. The fundamental IRUD scheme consists of six components: coordinating center (IRUD-CC), clinical center (IRUD-CL), clinical specialty subgroup (IRUD-CSS), analysis center (IRUD-AC), data center (IRUD-DC), and resource center (IRUD-RC). IRUD has registered many pedigrees with undiagnosed diseases, established their diagnoses with high diagnostic rate, identified novel causative genes and new disease entities, and promoted extensive data sharing and international collaboration. IRUD plays an important role in the national medical support network for rare and intractable diseases together with academic societies and national centers. Promotion of IRUD would be essential in elucidating causes and ultimately providing cures for rare and undiagnosed diseases.

## Introduction

With the recent progress of molecular genetics, causative genes of many diseases have been identified, and their therapeutic methods have been developed. However, there remain a substantial number of hereditary diseases whose causes are still unknown. According to Online Mendelian Inheritance in Man (OMIM) (URL: https://www.omim.org/), 9173 hereditary diseases have been registered as of August 2020, of which 3308 are diseases classified as phenotypes with unknown molecular basis. Even in diseases with known causative genes, it is often the case that the diseases are so rare and the clinical manifestations are so complicated that even exhaustive sets of ordinary diagnostic tests have never reached accurate diagnosis. These patients inevitably wander from hospitals to hospitals seeking correct diagnosis but eventually remain undiagnosed and untreated, which is the so-called “diagnostic odyssey.” Two surveys conducted by the Japan Agency for Medical Research and Development (AMED) have shown that there are more than 37,000 such cases, illustrating that diagnosing patients with such rare and undiagnosed diseases is still a major issue ^(1)^. In Japan, considerable achievements have been made in the research and countermeasures for intractable diseases, designated as “Nan-byo,” by the Ministry of Health, Labor and Welfare in Japan, which started in 1972 after the subacute myelo-optico neuropathy (SMON) pandemic in Japan. In 2015, the new intractable disease law was enforced to expand “Nan-byo” from 56 to 333 diseases, mandating further improvement in diagnostic accuracy of rare diseases.

With this background, the Initiative on Rare and Undiagnosed Diseases (IRUD) was launched as a research and development project with the aim of establishing a medical system for systematically diagnosing patients with rare and undiagnosed diseases and developing a system for collecting, accumulating, and sharing patient information and resources ^(2)^. IRUD for pediatric patients, IRUD-P, was launched in July 2015, followed by IRUD for adult patients, IRUD-A, in October 2015. In 2017, the two were integrated into one research project, IRUD (URL: https://www.amed.go.jp/en/program/IRUD/), the second phase started in 2018, and it has been steadily developing.

Research for rare and undiagnosed diseases has become a major international trend. Leading projects such as the Undiagnosed Diseases Project (UDP)/Undiagnosed Diseases Network (UDN) ^(3)^ in the United States, Genomics England ^(4)^ in the United Kingdom, and Finding of Rare Genes in Canada (FORGE) ^(5)^ have achieved unprecedented outcome. Furthermore, the International Rare Disease Research Consortium (IRDiRC) ^(6)^ has built a broader cooperation system, which AMED joined in 2015. One of the characteristics of IRUD compared to these preceding projects is that it incorporates Japan’s insurance medical care system so that research can be promoted efficiently at a much lower cost.

The inclusion criteria of “undiagnosed disease” in IRUD are as follows.

1. The patient remains undiagnosed for six months or longer (not necessarily for infants), and the symptom(s) affects his/her daily life, AND

2-1. There exist objective signs that cannot be reduced to a single organ; OR

2-2. There exists direct or indirect evidence of a genetic etiology as likely (e.g., similar symptom(s) found in the patient’s relatives) ^(2)^.

Here, an undiagnosed disease is clearly distinguished from an undetermined disease in which a clinical diagnosis has been made but the causative gene mutation has not been confirmed. For example, if spinocerebellar degeneration is clinically diagnosed but its causative gene has not been analyzed and the disease type has not been determined, then it is classified as an undetermined disease and excluded from IRUD.

## IRUD Structure/Function

**Figure 1.**
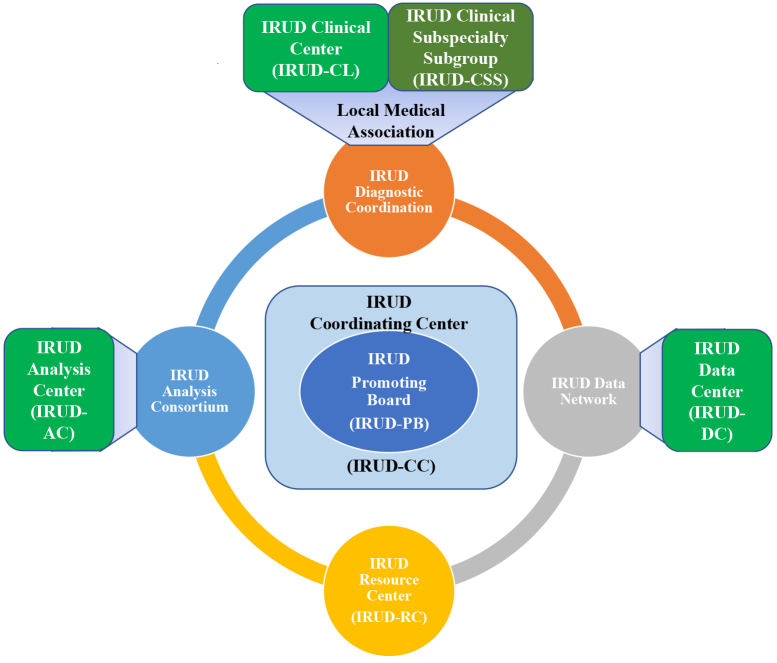
IRUD diagnostic scheme. IRUD diagnostic scheme consists of six components: IRUD Clinical Center (IRUD-CL), IRUD Clinical Specialists Subgroup (IRUD-CSS), IRUD Data Center (IRUD-DC), IRUD Analysis Center (IRUD-AC), IRUD Resource Center (IRUD-RC), and IRUD Coordination Center (IRUD-CC). IRUD-CC manages the IRUD Promotion Board (IRUD-PB), the highest decision-making organization. IRUD-CLs and IRUD-CSSs are integrated into IRUD Diagnostic Coordination, where community clinics/hospitals belonging to local medical associations participate. IRUD-CC temporally serves as IRUD-RC as well.

IRUD has successfully established a unified all-Japan diagnostic and research scheme for rare and undiagnosed diseases covering the entire geographic areas and specialty/subspecialty medical fields. The fundamental IRUD scheme consists of six components: coordinating center (IRUD-CC), clinical center (IRUD-CL), clinical specialty subgroup (IRUD-CSS), analysis center (IRUD-AC), data center (IRUD-DC), and resource center (IRUD-RC) (Figure 1 and Table 1).

**Table 1.** The IRUD Centers.

Components	Abbreviations	Institutions
IRUD Coordinating Center	IRUD-CC	National Center of Neurology and Psychiatry
IRUD Clinical Center	IRUD-CL	Sapporo Medical University
		Hokkaido University
		Asahikawa Medical University
		Akita University
		Tohoku University
		Chiba University
		Tokyo Medical & Dental University
		Tokyo University
		National Center for Child Health & Development
		Keio University
		Tokyo Women’s Medical University
		Tokyo Metropolitan Children’s Medical Center
		Kanagawa Children’s Medical Center
		Yokohama City University
		Niigata University
		Kanazawa University
		Shinshu University
		Yamanashi University
		Hamamatsu Medical University
		Nagoya University
		Nagoya City University
		Fujita Health University
		Osaka City University
		Osaka University
		National Cerebral & Cardiovascular Center
		Osaka Women’s & Children’s Hospital
		Kyoto University
		Kobe University
		Tottori University
		Kawasaki Medical School
		Hiroshima University
		Tokushima University
		Ehime University
		Nagasaki University
		Kumamoto University
		Ryukyu University
		National Center of Neurology and Psychiatry
IRUD Semi-Clinical Center	IRUD-SCL	Okinawa Prefectural Nanbu Medical Center & Children’s Medical Center
		Okinawa Child Development Center
		University of Tsukuba Hospital
		Kitasato University Hospital
		Saitama Medical University Hospital
		Kurume University Hospital
		Gunma Children’s Medical Center
		Dokkyo Medical University Hospital
		Aichi Developmental Disability Center
		Kurashiki Central Hospital
		Fukuoka University Hospital
		Saiseikai Yokohamashi Tobu Hospital
		Kagoshima City Hospital
		Jichi Children’s Medical Center Tochigi
		National Mie Hospital
IRUD Analysis Center	IRUD-AC	National Center for Child Health & Development
		Keio University
		Yokohama City University
		Nagoya University
		Osaka University
IRUD Data Center	IRUD-DC	Keio University
IRUD Resource Center	IRUD-RC	National Center of Neurology and Psychiatry

(1) IRUD Coordination Center (IRUD-CC): IRUD-CC administrates the whole system and strengthens governance by managing the IRUD Promotion Board (IRUD-PB), which is the highest decision-making organization. IRUD-CC also supervises and manages the progress of the entire research by conducting a regular survey twice a year. Furthermore, IRUD-CC manages a unified sample logistic system utilizing outsourcing contractors to facilitate the flow of samples and clinical information from IRUD-CLs to IRUD-ACs and simultaneously to centralize the information and sample repositories in IRUD-CC.

(2) IRUD Clinical Center (IRUD-CL): IRUD-CLs operate diagnostic committees that take charge of the process from the decision for patient entry based on the criteria for the establishment of final diagnosis upon receiving the analysis results from IRUD-AC. The diagnostic committee is composed of pediatricians and adult physicians of various specialties/subspecialties, clinical geneticists, genetic counselors, data scientists, and representative physicians from local medical associations to promote regional cooperation. The IRUD diagnostic committee in each IRUD-CL plays a central role in the regional diagnostic network with IRUD cooperative hospitals and medical associations referring IRUD entry candidates to IRUD-CL. In addition, semiclinical centers serve similar functions without funding, including diagnostic committees, to fill the geographic gaps of IRUD-CLs.

(3) IRUD Clinical Specialty/subspecialty Subgroup (IRUD-CSS): IRUD-CSSs are organized by assembling the members of diagnostic committees across the entire IRUD-CLs according to their specialties/subspecialties (Table 2). The roles of IRUD-CSSs are to support the activities of individual IRUD diagnostic committees and give advice from the professional standpoint of their specialties/subspecialties for the cases that cannot be resolved by themselves alone.

**Table 2.** IRUD Clinical Specialty Subgroups.

Category	Subcategory
Pediatrics	General
	Metabolic Diseases
	Congenital abnormality
Obstetrics	
Neurology	
Pulmonology	
Cardiology	
Gastroenterology	
Nephrology/Urology	
Endocrinology	
Hematology	
Allergy/Rheumatology	
Immunodeficiency	
Orthopedics	
Dermatology	
Ophthalmology	
Otorhinolaryngology	
Dentistry	
Psychiatry	
Clinical Genetics	
Local Medical Association	

IRUD-CLs and IRUD-CSSs are integrated into IRUD Diagnostic Coordination that realizes a comprehensive diagnostic scheme covering the entire geographic areas and specialty/subspecialty fields, where patients with any disease residing in any region can get access to IRUD.

(4) IRUD Analysis Center (IRUD-AC): IRUD-ACs receive clinical information and DNA samples from corresponding IRUD-CLs, conduct comprehensive genomic analysis centered on whole exome sequencing (WES), determine candidate causative mutations, and report to the IRUD-CLs. When causative mutations are undetermined, intensive research is conducted to identify novel causative genes/mutations by making full use of trio analysis, whole genome sequencing (WGS), multiomics analysis, and functional studies.

(5) IRUD Data Center (IRUD-DC): IRUD-DC operates IRUD Exchange, a data-sharing platform designed by incorporating Patient Archive system ^(7)^ that complies with the international standard phenotypic description system Human Phenotype Ontology (HPO) ^(8)^ and can be linked with the international data-sharing system MatchMaker Exchange ^(9)^. IRUD-Exchange promotes data sharing within IRUD researchers and serves as a gateway to international collaboration. Phenotypic and genomic information have been accumulated to promote the establishment of new causative genes and disease concepts.

(6) IRUD Resource Center (IRUD-RC): IRUD-RC establishes clinical information and DNA sample/cell line repositories and manages a utilization committee where application to utilize repositories are examined. IRUD-CC temporarily serves as the IRUD-RC.

In March 2020, the IRUD diagnostic scheme was composed of a total of 487 institutions consisting of 37 IRUD-CLs, 15 semiclinical centers, and 435 cooperative hospitals. Five IRUD-CLs also serve as IRUD-ACs, and one of them is also in charge of IRUD-DC. The National Center of Neurology and Psychiatry serves as IRUD-CC as well as IRUD-CL and has held IRUD-PB monthly. In March 2020, 21 IRUD-CSSs were organized with a total number of 469 clinical specialists to support the diagnostic committees in IRUD-CLs. IRUD-RC established resource repositories, including 3753 genomic DNA samples and 2512 lymphoblastic cell lines. Phenotypes and genetic data of 5609 pedigrees have been registered in IRUD Exchange, among which 62 were subjected to international data sharing.

**Figure 2.**
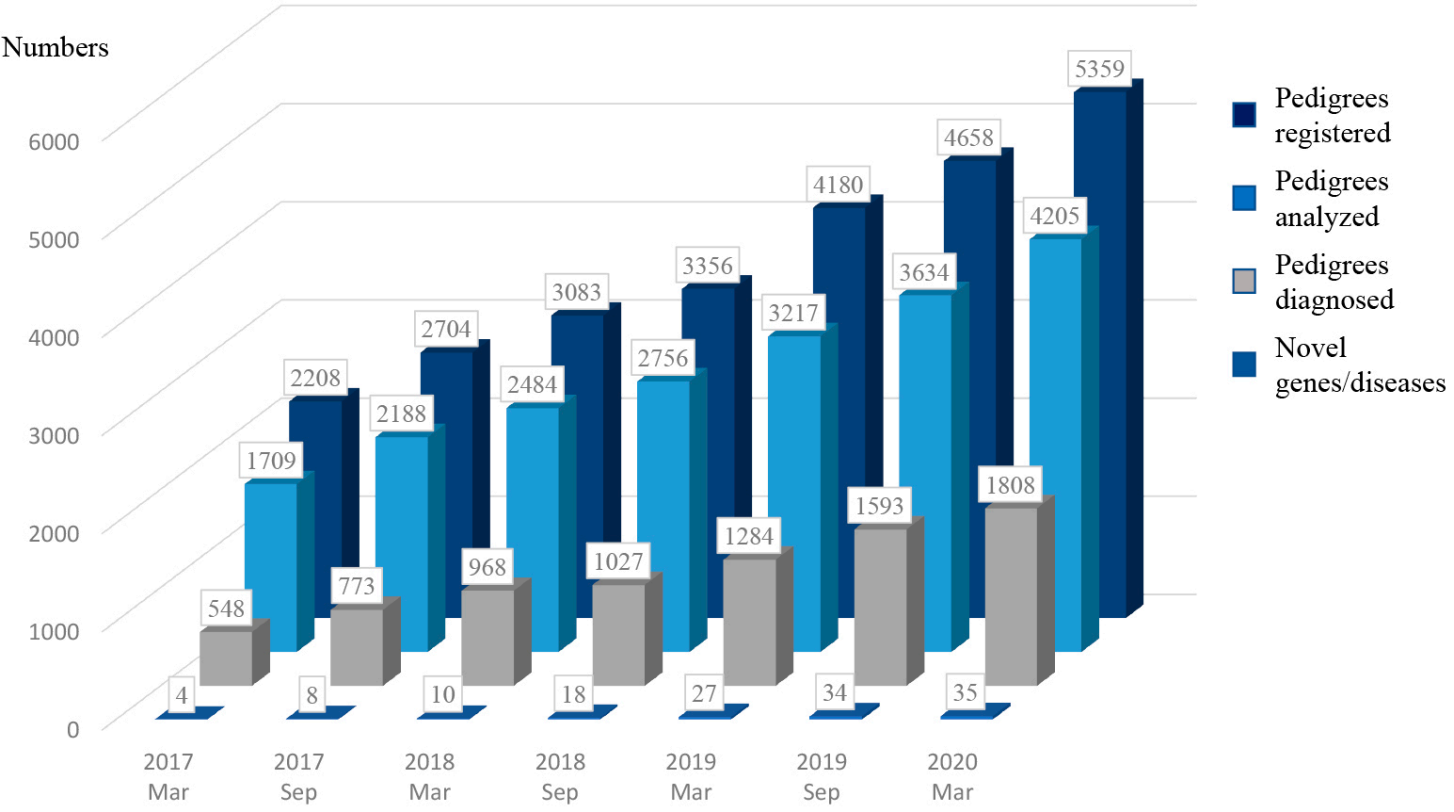
IRUD diagnostic yield. The cumulative number of pedigrees entered, pedigrees with analysis completed, and pedigrees with diagnosis established, and that of novel genes or disease entities are shown at the surveyed year and month.

In March 2020, 5359 pedigrees consisting of 15608 individuals were registered in IRUD. WES was completed in 4205 pedigrees, and final diagnosis was established in 1808 pedigrees (42.9%) (Figure 2). Thirty-five genes associated with novel disease entities or phenotypes have been discovered. The numbers of participants accepted, evaluated, and diagnosed have been larger than those of UDN [5369 vs 1845, 4205 vs 1457, and 1808 (42.9%) vs 434 (29.7%), respectively].

## “IRUD Beyond”- Advancing the Achievement of IRUD

“IRUD Beyond” has been launched as a pioneering research field for the purpose of further advancing the achievement of IRUD. “IRUD Beyond” consists of three conceptual frameworks: “beyond borders,” “beyond genotyping,” and “beyond diagnosis.” “Beyond borders” accelerates international data sharing of the research and development of IRUD more intensively. For this purpose, “Beyond borders” participated in Orphanet in 2017 and opened a website, Orphanet Japan Website, where five novel diseases or genes have been published as the achievement of IRUD. “Beyond genotyping” aims to further improve diagnostic accuracy by conducting functional studies of candidate mutations identified in IRUD, particularly utilizing small model animals such as nematodes, drosophila, and zebrafish so that mutant phenotypes can be obtained in a relatively short period. For this purpose, a model animal research coordinating network, Japan Rare Disease Models and Mechanisms Network (J-RDMM), was established in 2017, where a coordinating facility conducts matching between the candidate gene database and researcher registry database to find suitable researchers for the functional studies. “Beyond diagnosis” targets the development of innovative drug seeds based on pathogenic mutations identified in IRUD by adopting genome editing or induced pluripotent stem cells.

## The Perspectives of IRUD

IRUD has established an all-Japan comprehensive diagnostic system for rare and undiagnosed diseases covering the entire geographic areas and clinical specialties/subspecialties. IRUD plays an important role in the national medical support network for intractable diseases together with academic societies and national centers.

The network serves as an advisory organization in the diagnosis of and therapeutics for intractable diseases in prefectural intractable disease network hospitals, which are designated by prefectural governments and function as medical centers for intractable diseases. Most IRUD-CLs are also designated as prefectural intractable disease network hospitals, and the foundation for mutual cooperation has already been established. By effectively utilizing this IRUD diagnosis system, it is expected that the effectiveness of the intractable disease medical support network will be further enhanced and that it will contribute in improving the diagnosis accuracy of rare intractable diseases, elucidating the pathophysiology, and developing therapeutic methods. The IRUD diagnostic committee at each IRUD-C has established collaboration between pediatrician and adult physician of various specialties/subspecialties, which will contribute to the realization of transition medicine. In fact, undiagnosed diseases experienced by adult physicians often developed in childhood, and it is considered that the accuracy of the diagnosis will be further improved by cooperation between pediatricians and physicians for adults.

IRUD Exchange serves as a formatted phenotype/genotype database of rare diseases and potentially provides epidemiological evidence, including prevalence data, which is highly valuable for physicians, policy makers, and pharmaceuticals. Physicians can refer to the data in the daily clinical practice as a catalog of rare diseases in Japan. Policy makers can utilize the data to contrive the optimal solution to the supporting care systems covered by the health care insurance system for patients with rare and undiagnosed diseases. Pharmaceuticals have growing interest to obtain prevalence data of rare diseases to design strategic plans to develop orphan drugs. Thus, IRUD Exchange is considered a highly useful database for pharmaceutical companies aiming to develop therapeutic agents for rare and intractable diseases.

Identification of novel causative genes for rare and undiagnosed diseases and establishment of new disease entities do not merely have clinical significance of improving diagnostic accuracy. Obviously, the disease-causative genes define the most reliable and powerful pathogenic molecules. The identification of the novel causative genes is equivalent to the elucidation of the missing link between human genes and phenotypes in general. Without a thorough solution of the 3308 hereditary diseases classified as phenotypes with unknown causative genes based on OMIM database (August, 2020), complete understanding of human health and disease would be far from realization. More than 200 pedigrees in IRUD have individual candidate genes awaiting identification of additional pedigrees, offering an enormous opportunity for approaching toward this ultimate goal. Considering the characteristics of diseases with IRUD entry that present with multiple organ involvement, identification of the causative genes leads to elucidation of a common molecular mechanism in the formation, function, and maintenance of different organs. By identifying the “hub” of such molecular networks, it is expected that the hidden relationship in the organ-organ association in the normal function or disease state will be uncovered, allowing the development of innovative research by merging organ-specific findings.

Despite these spectacular accomplishments, there still remain more than 2000 pedigrees with undetermined causes. For further enhancement of diagnostic rate, IRUD has started to adopt the following cutting-edge strategies. Whole genome sequence analysis (WGS) would reveal intronic mutations such as noncoding repeat expansions or complex structural variations. RNA sequencing would identify aberrant splicing, transcriptional repression, exon skipping, or intron inclusion ^(10), (11)^. Proteomics or metabolomics analysis would reveal key mechanisms underlying the diseases suggesting the involvement of particular sets of genes ^(12)^. For such purposes, a lymphoblast cell line repository in IRUD is considered highly valuable and has already been vigorously utilized. This integrative multiomics approach would break through to the next level of genomic medicine for rare and undiagnosed diseases.

## Conclusion

IRUD has realized a nationwide comprehensive genetic diagnosis scheme for rare and undiagnosed diseases and succeeded in establishing diagnosis and identifying novel causative genes and new disease entities. The high diagnostic rate in IRUD has testified that genetic examination plays a pivotal role in the medical services of rare and undiagnosed diseases. Further promoting IRUD with extensive international collaboration would be essential in elucidating causes, improving quality of life, and ultimately providing cures for patients with rare and undiagnosed diseases.

## Article Information

### Conflicts of Interest

None

## References

[1] Adachi T, Imanishi N, Ogawa Y, et al. Survey on patients with undiagnosed diseases in Japan: potential patient numbers benefiting from Japan’s initiative on rare and undiagnosed diseases (IRUD). Orphanet J Rare Dis. 2018;13(1):208. 30458817 10.1186/s13023-018-0943-y PMC6245805

[2] Adachi T, Kawamura K, Furusawa Y, et al. Japan’s initiative on rare and undiagnosed diseases (IRUD): towards an end to the diagnostic odyssey. Eur J Hum Genet. 2017;25(9):1025-8. 28794428 10.1038/ejhg.2017.106 PMC5558173

[3] Gahl WA, Mulvihill JJ, Toro C, et al. The NIH undiagnosed diseases program and network: applications to modern medicine. Mol Genet Metab. 2016;117(4):393-400. 26846157 10.1016/j.ymgme.2016.01.007 PMC5560125

[4] Turnbull C. Introducing whole-genome sequencing into routine cancer care: the Genomics England 100 000 Genomes Project. Ann Oncol. 2018;29(4):784-7. 29462260 10.1093/annonc/mdy054

[5] Beaulieu CL, Majewski J, Schwartzentruber J, et al. FORGE Canada Consortium: outcomes of a 2-year national rare-disease gene-discovery project. Am J Hum Genet. 2014;94(6):809-17. 24906018 10.1016/j.ajhg.2014.05.003 PMC4121481

[6] Ayme S. IRDiRC-recommended. Eur J Hum Genet. 2016;24(7):955. 27307111 10.1038/ejhg.2015.236 PMC5070888

[7] Groza T, Kohler S, Doelken S, et al. Automatic concept recognition using the human phenotype ontology reference and test suite corpora. Database. 2015;2015. 10.1093/database/bav005 PMC4343077 25725061

[8] Groza T, Kohler S, Moldenhauer D, et al. The human phenotype ontology: semantic unification of common and rare disease. Am J Hum Genet. 2015;97(1):111-24. 26119816 10.1016/j.ajhg.2015.05.020 PMC4572507

[9] Buske OJ, Schiettecatte F, Hutton B, et al. The matchmaker exchange API: automating patient matching through the exchange of structured phenotypic and genotypic profiles. Human Mutat. 2015;36(10):922-7. 10.1002/humu.22850 PMC4775166 26255989

[10] Gonorazky HD, Naumenko S, Ramani AK, et al. Expanding the boundaries of RNA Sequencing as a diagnostic tool for rare Mendelian disease. Am J Hum Genet. 2019;104(3):466-83. 30827497 10.1016/j.ajhg.2019.01.012 PMC6407525

[11] Cummings BB, Marshall JL, Tukiainen T, et al. Improving genetic diagnosis in Mendelian disease with transcriptome sequencing. Sci Transl Med. 2017;9(386). 10.1126/scitranslmed.aal5209 PMC5548421 28424332

[12] Roos A, Thompson R, Horvath R, et al. Intersection of proteomics and genomics to “Solve the Unsolved” in rare disorders such as neurodegenerative and neuromuscular diseases. Proteomics Clin Appl. 2018;12(2):1700073. 10.1002/prca.201700073 29059504

